# Silicon Photomultiplier Sensor Interface Based on a Discrete Second Generation Voltage Conveyor

**DOI:** 10.3390/s20072042

**Published:** 2020-04-05

**Authors:** Vincenzo Stornelli, Leonardo Pantoli, Gianluca Barile, Alfiero Leoni, Emanuele D’Amico

**Affiliations:** Department of Industrial and Information Engineering and Economics (DIIEE), University of L’Aquila, Piazzale Pontieri 1, Monteluco di Roio, I 67100, 67100 L’Aquila, Italy; leonardo.pantoli@univaq.it (L.P.); gianluca.barile@univaq.it (G.B.); alfiero.leoni@univaq.it (A.L.); emanuele.damico@student.univaq.it (E.D.)

**Keywords:** current mode, sensor interface, silicon photomultiplier, transimpedance amplifier, voltage current conveyor

## Abstract

This work presents the design of a discrete second-generation voltage conveyor (VCII) and its capability to be used as electronic interface for silicon photomultipliers. The design addressed here exploits directly at the transistor level, with commercial components, the proposed interface; the obtained performance is valuable considering both the discrete elements and the application. The architecture adopted here realizes a transimpedance amplifier that is also able to drive very high input impedance, as usually requested by photons detection. Schematic and circuital design of the discrete second-generation voltage conveyor is presented and discussed. The complete circuit interface requires a bias current of 20 mA with a dual 5V supply voltage; it has a useful bandwidth of about 106 MHz, and considering also the reduced dimensions, it is a good candidate to be used in portable applications without the need of high-cost dedicated integrated circuits.

## 1. Introduction

Discrete components are still the primary solution for many electronic applications [1]. When a low integration factor can be adopted or system dimensions are not critical, hybrid circuits can still obtain good performance and synthesize the desired electrical behavior, because, from a technology point of view, commercially available electronic components are able to provide almost state-of-the-art performances. Additionally, in integrated systems, the capability to design and test preliminary hybrid solutions is usually beneficial for the designer in order to validate the circuit functionality [2,3].

From different points of view, silicon photomultipliers (SiPMs) are becoming an enabling technology in several fields (physical, medical, automotive applications, etc.) replacing the traditional photomultiplier tubes, especially in portable sensor applications. The technology advancements in recent times have achieved a high sensitivity and detection capability in compact dimensions and also require a reduced power consumption. Even though SiPMs represent a new powerful solution in the realization of photon detectors, on the one hand, on the other hand, they stress the electronics, requiring advanced interfaces with strict performances. This is due to the large parasitics provided by the SiPM, in particular, the output capacitance that increases even more when considering SiPM arrays, a typical solution adopted when a larger sensitive area is required.

In the literature, some SiPM sensor interfaces have been presented in the last few years [4,5,6,7], and this topic is currently a sensible task due to a large number of possible applications, as previously said. These works usually present electronic solutions at an integrated level, and they are mainly based on a voltage-design approach [8,9,10,11]. To the best of our knowledge, no hybrid solutions have been presented for this purpose for practical applications. In addition, previously presented works usually present voltage-mode design, because it is preferable for noise performance, even if it lacks velocity with respect to current-mode circuits [12]. Only a few solutions in mixed voltage/current mode have already been presented in the literature [13,14,15,16], the latter proposed by the same authors [15,16].

The discrete, full commercial component-based sensor interface proposed here achieves many benefits. First of all, it is a mixed voltage-mode current-mode design, and thanks to this choice, it is able to merge the two different design conceptions, taking advantage of both of them. In addition, it is the first discrete hybrid interface designed at the transistor level, making use of the so-called second-generation voltage conveyor (VCII) [17,18,19,20]. To the best of our knowledge, no other discrete VCIIs have been presented in the literature up to now. The proposed circuit was fabricated, and a smart prototype board was tested in our laboratory with promising results that are reported here, providing a feasible demonstration of its usability in practical applications. In order to test the interface, the sensor was emulated by means of an equivalent circuit as discussed in this paper, allowing us to easily mimic different working conditions.

In the following, Section 2 summarizes and makes some remarks on the VCII characteristics, Section 3 illustrates the proposed solution and describes the achievable performances, while Section 4 presents measurement results and the overall characteristics of the sensor interface. Finally, in Section 5, conclusions are drawn.

## 2. VCII Characteristics

**Figure 1:**
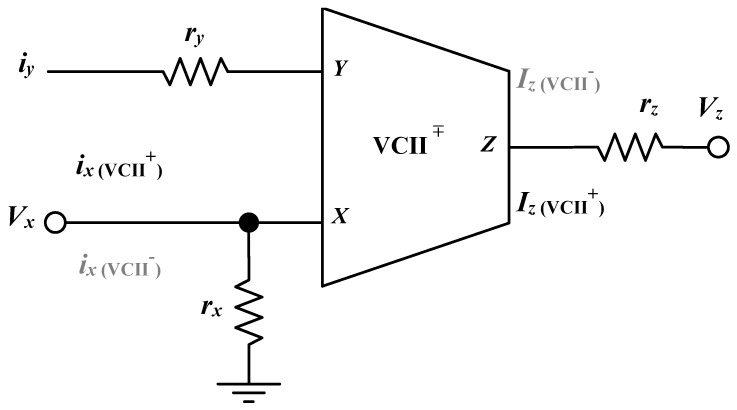
Block representation of the second-generation voltage conveyor (VCII) with its parasitic port impedances.

The VCII is a three-port device, presented for the first time in [17] and conceived for duality from the current conveyor [21,22,23]. It has two input ports, *X* and *Y*, and an output port *Z*, as shown in Figure 1. It is logically representable as a current buffer between *Y* and *X* terminals and a subsequent voltage buffer between the *X* and *Z* ports. The peculiarity of the VCII that makes it suitable for the proposed application is that it inherently acts as a transimpedance amplifier between the *Y* and *Z* terminals, obtaining at the same time low input and output impedances. The relationship between external terminals can be summarized as in Equation (1), where *α* is the voltage gain between the *X* and *Z* ports and *β* is the current gain between *Y* and *X* terminals, while *r_x_*,*_y_*,*_z_* are the parasitic impedances relative to the corresponding *X*, *Y*, and *Z* terminals. Ideally, *α* and *β* are equal to one (*β* in absolute value) and *r_x_* is infinite, while *r_y_* and *r_z_* are equal to zero.

(1) $$\left[\begin{array}{c} i_{x}\\ v_{y}\\ v_{z} \end{array}\right]=\left[\begin{array}{ccc} \frac{1}{r_{x}} & \pm \beta & 0\\ 0 & r_{y} & 0\\ \alpha & 0 & r_{z} \end{array}\right]\left[\begin{array}{c} V_{X}\\ I_{Y}\\ I_{Z} \end{array}\right]$$

**Figure 2:**
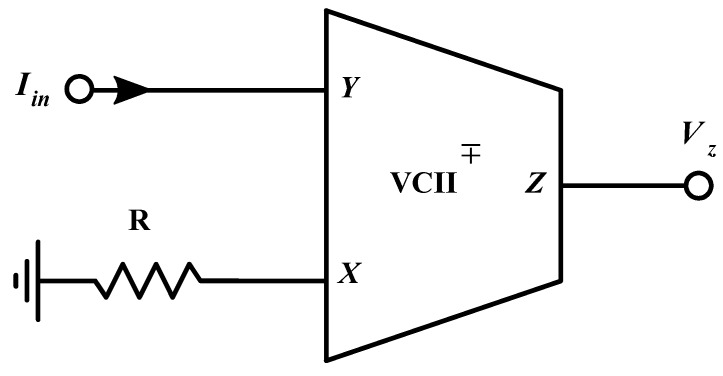
VCII configuration as current to voltage converter.

Further advantages of this building block are the capability to ensure faster operation with respect to traditional purely voltage-mode circuits, to maintain good performance up to a relatively high frequency, and to provide a constant transimpedance gain regardless of the operational bandwidth. The latter assumption can be justified by considering and discussing the configuration proposed in Figure 2. Assuming that *r_x_* >> *R*, and by considering Equation (1), we have the following:

(2) $$V_{x}=I_{x}R=\pm \beta I_{in}\left(Rr_{x}\right)≅\pm \beta I_{in}R$$

Then, combining Equation (2) with Equation (1), the output voltage at the *Z* terminal can be obtained as in Equation (3):

(3) $$V_{out}=V_{z}=\alpha V_{x}=\pm \beta \alpha RI_{in}$$

and so, the final version of the transimpedance transfer function, *F_TI_* (which is the transimpedance gain) is synthesized as follows:

(4) $$F_{TI}=\frac{V_{out}}{I_{in}}=\pm \alpha \beta R$$

As evident from Equation (4), the gain linearly depends only on the value of external resistor *R*, disregarding the parameters *α* and *β* that are almost equal to one, if the circuit is properly designed.

## 3. Transistor-Level Hybrid Interface

**Figure 3:**
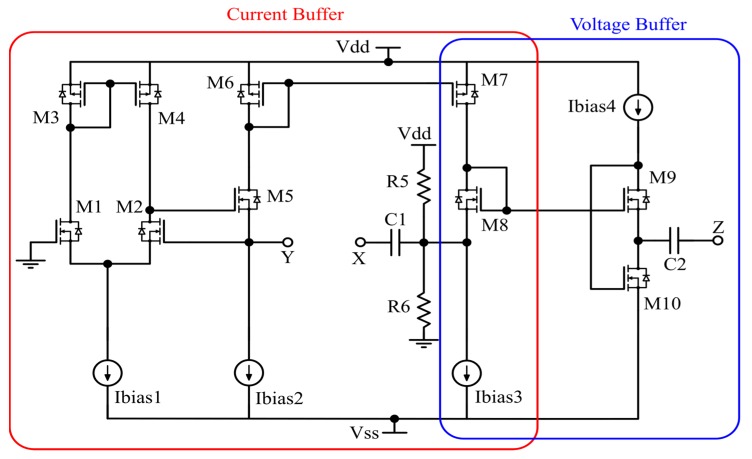
Simplified schematic of the proposed discrete VCII.

The sensor interface proposed here is realized with the described VCII acting as a transimpedance amplifier and implemented completely at the transistor level with discrete components. The simplified schematic of the proposed VCII is reported in Figure 3.

The current sources are physically implemented with current mirrors whose reference current is obtained by the provided bias voltage. The input stage is the so-called regulated common-gate [8,24], in which the traditional common source amplifier is implemented by a differential amplifier, whose differential pair consists of the transistors *M*_1_ and *M*_2_. The *Y* terminal of the VCII is considered to be the inverting input of the differential pair, while the non-inverting input is grounded; in this way, better rejection of the common-mode DC input noise is achieved. The input current incoming from the *Y* terminal is then mirrored on the *X* terminal with the current mirror implemented by *M*_6_ and *M*_7_ and the current sources *I_bias_*_2_ and *I_bias_*_3_. In this manner, the input current buffer is realized between the *Y* and *X* terminals.

Conversely, the output section of the schematic implements the voltage mirroring between the *X* and *Z* ports. The voltage buffer is obtained by the so-defined flipped voltage follower (FVF), which, compared to a traditional common-drain circuit, realizes a feedback on the bias line of the output transistor *M*_9_. In this way, it is possible to obtain a constant bias current and thus a fixed drain-source voltage for this transistor, achieving the desired voltage buffering action. Regarding the stability design criterion of the FVF block, we have taken into account the already developed theory reported in [25]. The current buffer block design also follows general stability consideration, because the two blocks are cascaded and there is no feedback loop between them. In addition, in assessing the noise impact, the designer should consider both current and voltage noise contributions. In the proposed front-end circuitry, the main noise contributions are due to the two bias currents Ibias2 and Ibias3, as well as the flipped voltage follower-biasing architecture.

**Figure 4:**
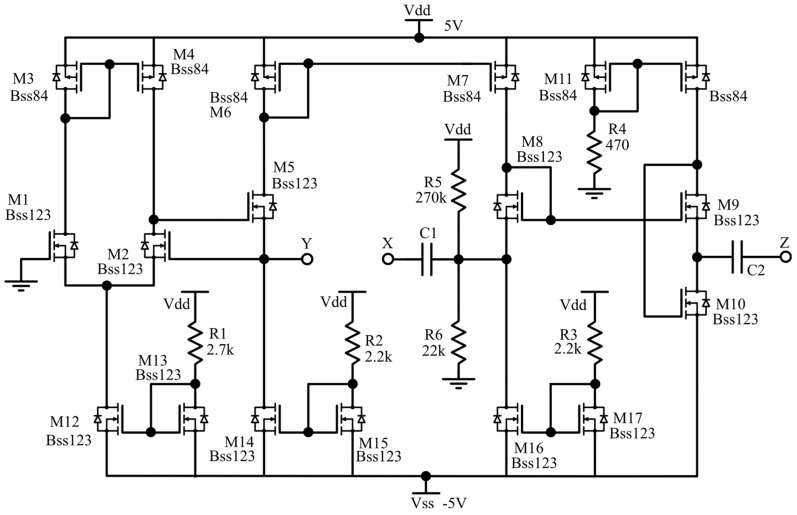
Complete schematic of the defined VCII in SPICE Environment.

For completeness, in Figure 4 the complete schematic of the proposed VCII is depicted, where the resistors *R*_1_ and *R*_2_ are used to properly bias the transistor *M*_8_, while *C*_1_ and *C*_2_ are AC coupling capacitors.

By considering the proposed VCII as a transimpedance amplifier, it is possible to evaluate also input and output impedances that can be calculated with Equations (5) and (6), respectively.

(5) $$R_{in}=\frac{1}{g_{m}\left(M_{5}\right)g_{m}\left(M_{2}\right)\left(r_{o}\left(M_{2}\right)//r_{o}\left(M_{4}\right)\right)}$$

(6) $$R_{out}=\frac{1}{g_{m}\left(M_{9}\right)g_{m}\left(M_{10}\right)r_{o}\left(M_{10}\right)}$$

**Figure 5:**
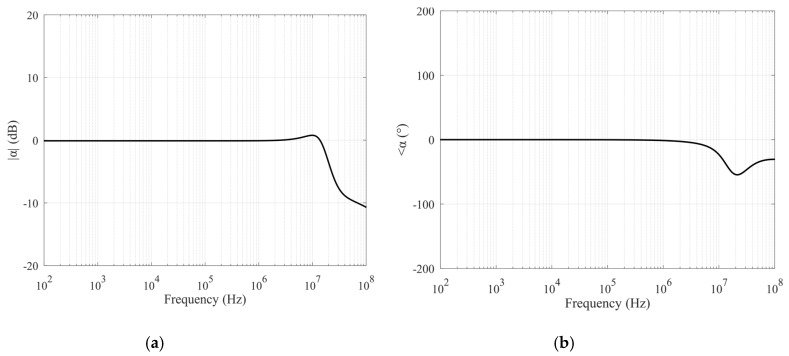
Voltage buffer characteristics: magnitude (**a**) and phase (**b**) of *α*.

**Figure 6:**
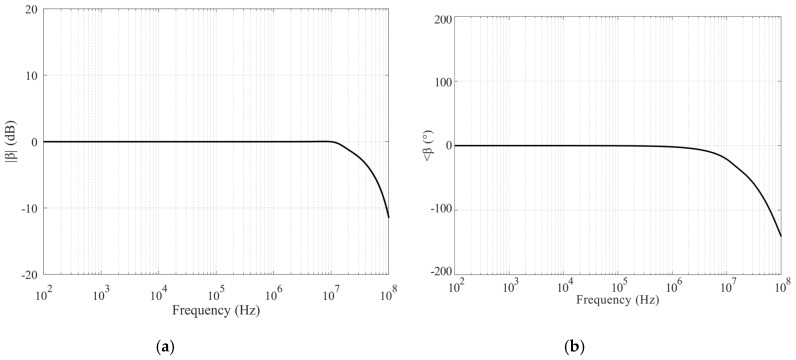
Voltage buffer characteristics: magnitude (**a**) and phase (**b**) of *β*.

The circuit was simulated in LTSPICE Environment and completely realized by using discrete metal-oxide semiconductor (MOS) transistors. The selected devices are the N-channel BSS123 and the P-channel BSS84, both from ON Semiconductor. They have fast switching performances and are particularly suited for low-voltage and low-current applications. The complete schematic of the circuit is reported in Figure 4. Performances of the VCII have been optimized by varying current and bias voltage and by analyzing *α* and *β* parameters with the goal to make them close to unity in the largest possible bandwidth. Simulated results are shown in Figure 5 and Figure 6, respectively; they demonstrate the capability of the proposed circuit to effectively work even at very high frequencies thanks to the large bandwidth. In more detail, Figure 5a,b shows the magnitude and phase of the transfer function of the voltage buffer section of the interface, demonstrating for *α* a -3dB bandwidth of about 55 MHz; while Figure 6a,b illustrates the characteristics of the circuit section operating as a current buffer: the −3dB bandwidth for the *β* parameter is about 33 MHz. 

**Figure 7:**
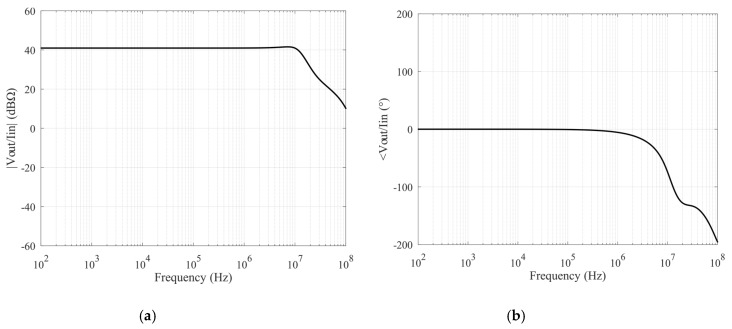
Transimpedance characteristics: magnitude (**a**) and phase (**b**).

By connecting an external resistor on the *X* terminal of the described VCII (as shown in Figure 2) and considering the overall transfer function between *Y* and *Z* terminals, the transimpedance characteristic can be evaluated and optimized, it being the main design parameter in order to maximize the performances for the proposed interface in term of bandwidth and response time. Figure 7a,b shows the complete transfer function *V_out_*/*I_in_* as magnitude and phase charts, respectively. They have been obtained considering a 100 Ω resistive load on the *X* terminal, even if, as will be better discussed later, this component will determine mainly the gain value of the interface. Even so, in these operating conditions, the results clearly show an almost constant gain of 42 dB and a useful bandwidth of about 106 MHz.

## 4. SiPM Description

**Figure 8:**
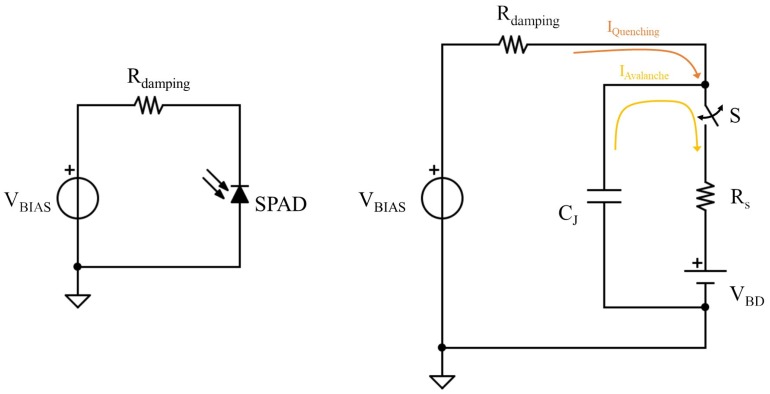
Silicon photomultiplier (SiPM) equivalent circuit.

In Figure 8 (left side), a simplified circuital representation of a single SiPM elementary cell is depicted. It is mainly composed of a single-photon avalanche diode (SPAD), in series with a damping resistor *R_DAMPING_*. The single cell, also called a pixel, is connected in parallel with other identical structures, with a common electrode structure, forming a multi-pixel photon counter matrix. The SPAD here is intended to operate in reverse bias, setting the supply voltage *V_BIAS_* above the breakdown threshold of the photodiode. Consequently, the SPAD enters into a very unstable state of operation, called Geiger mode, which represents the core of the sensor functionality. In this state, when a single photon hits the SPAD sensitive area, its energy is transferred to an electron–hole couple generation, which starts a chain reaction for which, other electron–hole couples are created because of the high electric field imposed by the external reverse bias voltage. This leads to the formation of a high current flow, which, however, is limited by the damping resistor, which quenches the self-sustained phenomenon, thus restoring the Geiger state. From a circuital point of view (Figure 8, right side), the SPAD can be described as a small resistance *R_S_* in series with a voltage generator, which represents the diode breakdown voltage, in parallel with the junction Capacitor *C_J_*. The switch *S* is introduced to simulate the occurrence of a photon in the sensitive area.

At regime, the capacitor *C_J_* is charged at *V_BIAS_*, and the Geiger mode is active. When a single photon is absorbed by the pixel, the switch *S* closes, and the junction capacitance starts discharging quickly through the resistor *R_S_*. As the capacitor discharges, the voltage decreases towards zero and is restored by the external bias voltage through the damping resistor, thus quenching the avalanche effect. As a final result, this process produces a current pulse, where the rise time is led by the diode equivalent series resistance *R_S_*, having a time constant *τ* = *R_S_C_J_*, while for the fall time, the main contribution is given by the damping resistor, having a time constant of *τ* = *R_DAMPING_*·*C_J_*. Therefore, the peak value of the current pulse *I_peak_* can be computed as follows:

(7) $$I_{peak}=\frac{V_{BIAS}-V_{BD}}{R_{DAMPING}+R_{s}}$$

and it can be modified only by changing the bias voltage value *V_BIAS_*, with the other contributions fixed by the SiPM technology. If this single pixel is connected in parallel with other identical cells, it is clear that the corresponding total parasitic capacitance *C_TOT_* of the SiPM is equal to the following:

(8) $$C_{TOT}=NC_{J}$$

where *N* is the number of pixels connected in parallel. Therefore, the total capacitance could be large, up to thousands of pF, depending on the SiPM model and the number of cells constituting the sensor matrix, and this represents the most critical aspect for a front-end circuitry, because it can considerably degrade the interface bandwidth, if not properly designed. In the proposed VCII-based solution, the input impedance $R_{in}$ at the Y node, as shown in Equation (5), is considerably less than 1. Therefore, the parasitic capacitance of the SiPM, expressed as $1/(s\cdot C_{TOT})$, will be greater than the transimpedance amplifier input impedance even at higher frequencies, ensuring a large bandwidth.

## 5. Results and Measurements

**Figure 9:**
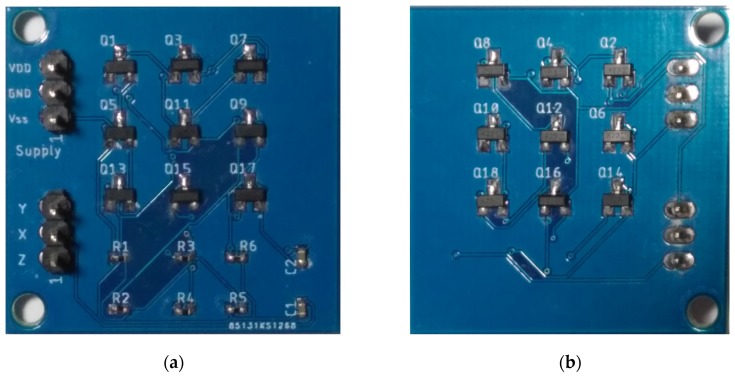
Discrete prototype board: top (**a**) and bottom (**b**) views.

The sensor interface that has been designed and discussed in Section 3 was fabricated as a discrete prototype. The complete schematic, designed in LTSPICE Environment, was optimized at the layout level through Autodesk Eagle software. The circuit was organized in a compact double-sided printed circuit board (PCB); the transistors NMOS BSS123 and PMOS BSS84 have a SOT23 package, while for passive components, a 0402-inch socket was chosen. The substrate was FR4 with a 0.7 mm thickness, and the board size was 40 mm × 40 mm. Figure 9a,b shows the top and bottom sides of the realized prototype board.

**Figure 10:**
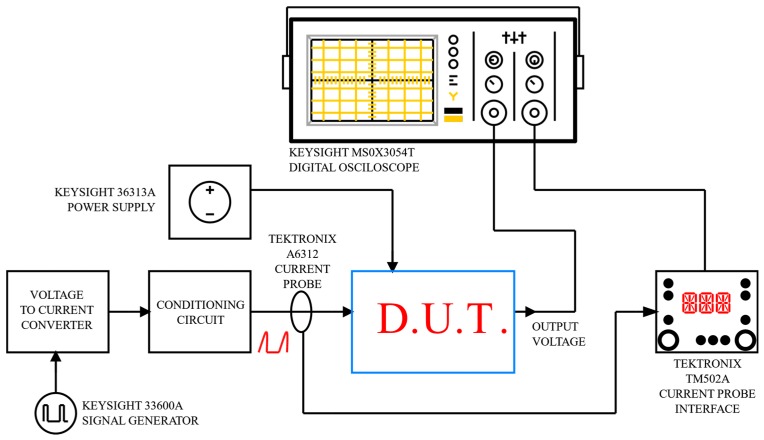
Test bench scheme of the prototype board.

The interface was tested both in time and frequency domains for complete characterization. The stimulus for time-domain measurements was generated by using the Keysight 33600A Signal Generator, which can define a single pulse or a pulse train with a shape factor and characteristics similar to that provided by a typical SiPM sensor [26]. Both the time domain and frequency domain measurements were performed utilizing the digital oscilloscope and signal analyzer InfiniiVision MS0X3054T provided again by Keysight Technologies. DC power supply and current probes (Keysight E36313A) were also used. In order to emulate real SiPM electric behavior, a simple conditioning circuitry was added between the signal generator and the proposed front-end system. In particular, the generated voltage pulses were converted into current pulses by means of a resistor, while the produced current signal was then buffered by means of the commercial current buffer AD844 from Analog Devices. Finally, a signal conditioning circuit with a 320 pF shunt capacitor was added at the output of the current buffer to reproduce the sensor parasitic capacitance effect. The characteristics of the commercial SiPM S13360-3050 from Hamamatsu were considered as a reference for the signal conditioning circuit definition. In Figure 10, a block diagram of the complete test bench used for measurements is reported. The device under test (D.U.T.) block refers to the proposed sensor interface, whose schematic is shown in Figure 4.

**Figure 11:**
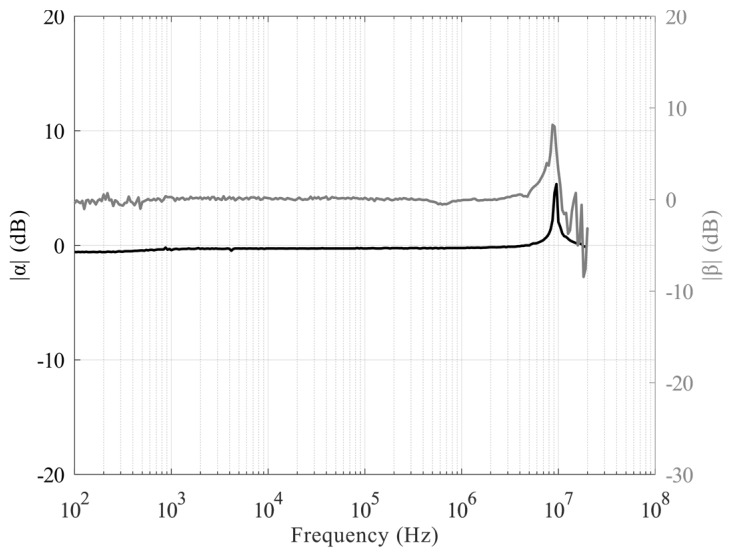
Measured transfer function in magnitude of both *α* (left axis) and *β* (right axis).

At first, the functionalities of the base building block, the VCII shown in Figure 3, were tested. Both α and β characteristics were evaluated by considering a voltage/current input signal, respectively. The analysis was done by setting a relatively small amplitude, thereby preserving the circuit operation in the linear regime, and both were kept at a fixed and variable frequency. In this manner, both the values and bandwidths of these parameters were evaluated directly in the time domain, and the data confirmed the simulation results with good accuracy. The operational bandwidth of α and β are reported in Figure 11. They were obtained by sweeping the frequency of the input source and iterating the measurements each time. The transimpedance characteristics were also verified with a circuital setup organized as in Figure 2.

**Figure 12:**
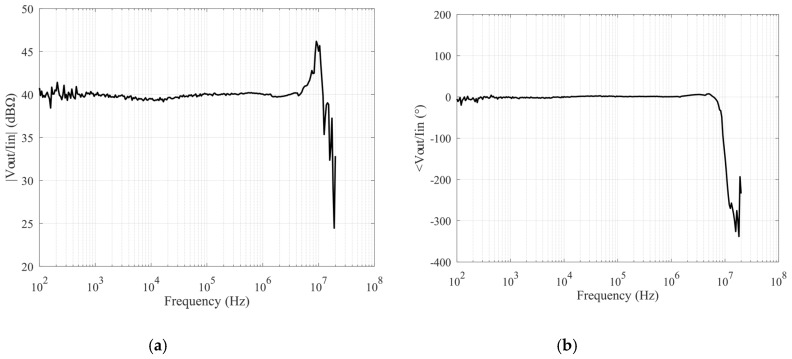
Measured transimpedance characteristics: magnitude (**a**) and phase (**b**).

**Figure 13:**
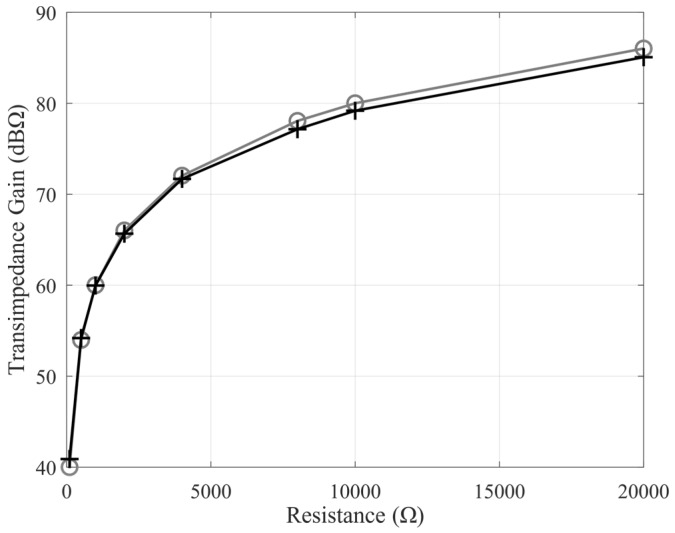
Ideal (grey trace) and measured (black trace) transimpedance gain at different values of the external X terminal gain resistor.

The transimpedance gain remains constant at a value of value 39.8 dB, while the operation bandwidth is 150 MHz when considering an external 100 Ω resistor on the X terminal. The achieved measurements are shown in Figure 12. Comparing them with simulations (Figure 7), there is good agreement, also confirming the accuracy of the spice models of the active devices beyond the feasibility of the design. In addition, by changing the cited resistor, it is possible to modify the transimpedance gain, as shown in Figure 13. This is a further advantage that justifies the use of the proposed architecture.

**Figure 14:**
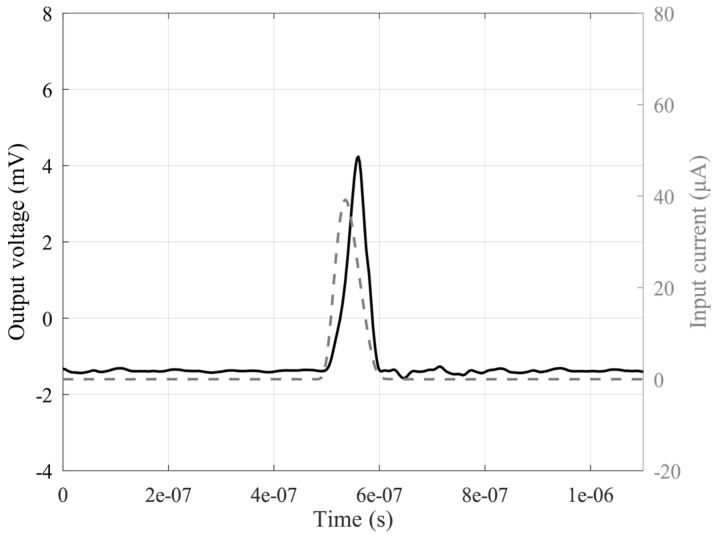
Input current pulse with a duration of 80 ns (grey trace, right axis) and output voltage signal of the defined SiPM interface (black trace, left axis).

**Figure 15:**
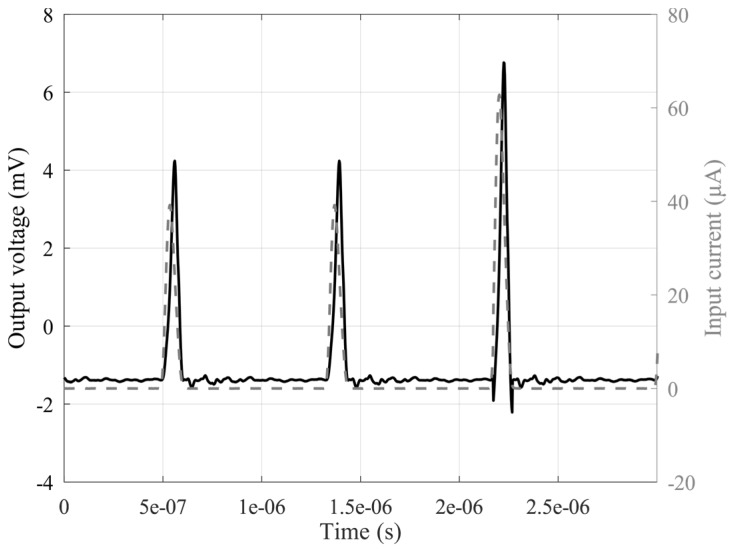
Output voltage signal of the circuit (black trace, left axis) when considering a current pulse train with different amplitudes and repetition time (grey trace, right axis).

Finally, the interface was also tested with respect to short pulses or pulse trains. So, time-domain signals reproducing the real signals coming from SiPMs were defined by using the aforementioned signal generator. Pulses of different duration were considered, and the results demonstrate the capability of the circuit to properly detect and follow short pulses up to 80 ns, as shown in Figure 14, while in Figure 15, an incoming pulse train at different amplitudes is shown together with the output signal. As reported, the proposed circuit shows good sensitivity and a good settling time at different amplitude levels, making it possible to use the interface in real-world applications when a fast detection of several pulses in a short time is a typical circumstance. In Table 1, a comparison with different literary solutions is reported. It is worth noting that the solution we have proposed, to the best of our knowledge, is the first transistor-only VCII discrete interface that can be used in practical applications with silicon photomultipliers. As a result, we have been able to include in Table 1 only equivalent integrated solutions. Some transimpedance amplifiers in both CMOS and SiGe technologies are shown. 

Obviously, our work has a large power consumption because it is realized with hybrid components and the power consumption is strictly dependent on the technology and the required biasing conditions. It is worth noting that in many applications, the power consumption is not critical, and the sensor interface minimally affects the total power consumption of the complete system. It is also important to remark that the main novelties are the realization of a functional interface with discrete elements, the ease of realization, and the very low cost. To conclude, even though we present a discrete solution, our results are comparable with integrated interfaces, so the overall performance of the proposed solution is credible.

## 6. Conclusions

In this paper, a fully discrete MOS transistor-only VCII was presented for the first time. It was designed, realized with commercial components, and tested with successful results. The capability to use the proposed circuit as a sensor interface for silicon photomultipliers was also evaluated and tested. The proposed solution has compact dimensions and low power consumption and is able to provide an agile response for quick incoming signals. The system was tested with both short current input pulses and pulse trains, reproducing the operative conditions of SiPM systems. As shown, the proposed interface provides stable characteristics, demonstrating its feasibility to be used in practical applications.

## Figures and Tables

**Table 1:** Comparison with different literary solutions.

Refs.	Technology	Supply	Power	Transimpedance Gain	Bandwidth	Noise
[8]	CMOS 130 nm	1.2 V	0.34 µW	100 dB	10 MHz	2.7 mVrms (output)
[27]	CMOS 350 nm	3.3 V	0.68 µW	100 dB	50 MHz	1300 e- (ENC)
[28]	CMOS 350 nm	3.3 V	0.68 µW	500	150 Hz	2 uVrms (input)
[29]	CMOS 350 nm	3.3 V	0.68 µW	/	/	6.9 mVrms (output)
[30]	SiGe 130 nm	−3.2 V	82 µW	56 dB	45 GHz	$30.6\;\mathrm{pA}/\sqrt{Hz}$
This work	Discrete	±5 V	200 mW	42 dB	106 MHz	9 mV_RMS_ (output)
